# AIP-based Professional Intervention Program for Adversity for trauma and stress reduction in groups: a pilot study in Ethiopia

**DOI:** 10.3389/fpsyt.2024.1351713

**Published:** 2024-03-20

**Authors:** Solomon Woldemariam, Dorothy Ashman, Esly Carvalho, Sik-Lam Wong, Raquel Hoersting

**Affiliations:** ^1^ Hasset Psychotherapy and Training Center, Addis Ababa, Ethiopia; ^2^ Retired, Lewisburg, PA, United States; ^3^ Trauma Clinic, Brasilia, Brazil; ^4^ Department of Social Work, California State University, Hayward, CA, United States; ^5^ Psychology Department, University of Prince Edward Island, Charlottetown, PE, Canada

**Keywords:** PIPA, professional intervention program for adversity, EMDR therapy, flash technique, pillars of life, trauma, cross-cultural psychological interventions, group trauma work

## Abstract

**Introduction:**

Drawing from the principles of EMDR (Eye Movement Desensitization and Reprocessing) therapy and the AIP model, the Professional Intervention Program for Adversity (PIPA) was developed with the objective of amalgamating low-intensity group exercises into a unified framework, as a comprehensive intervention for group therapy. The PIPA Program integrates various aspects of EMDR therapy—such as stabilization, resourcing, desensitization, reprocessing, and forming beliefs about the self and future—into a cohesive program. The program’s structure includes self-regulation exercises, the Pillars of Life, the Flash Technique, and the Quadrants exercise.

**Methods:**

The PIPA Program was administered to more than 220 individuals with a high probability of traumatization by the two-year civil war in Ethiopia (2020-2022).

**Results:**

The results of this study show a statistically significant improvement in PTSD symptoms on PCL-5 scores (from M = 38.58 to M = 20.59) after completing the entire PIPA Program and statistically significant lower SUDS scores within the program segments of the Flash Technique and the Quadrants exercises.

**Discussion:**

Future studies should explore the long-term effects of the PIPA Program and its broader application across different therapeutic contexts. The findings suggest that the PIPA Program is a promising group-based intervention for trauma treatment that is safe and effective, especially in non-clinical settings and for culturally diverse populations.

## 1 Introduction

Eye Movement Desensitization and Reprocessing (EMDR) therapy is an evidence-based psychotherapeutic approach for the treatment of Post-Traumatic Stress Disorder (PTSD) and other trauma-based symptoms (1). The Adaptive Information Processing model AIP, which is the underpinning theoretical framework of EMDR therapy, posits that trauma-related symptoms arise when disturbing experiences overwhelm the brain’s natural ability to process and integrate information. Memories associated with traumatic events may be inadequately processed and stored in a dysfunctional manner, which can lead to the persistence of negative emotions, physical sensations, and beliefs related to the traumatic experience, and can contribute to the development of a range of psychological symptoms and distress (2).

The current version of the Professional Intervention Program for Adversity (PIPA) was developed by Carvalho and Guz in 2022 with the objective of amalgamating low-intensity group exercises into a unified framework, as a comprehensive intervention for group therapy. The PIPA Program integrates various aspects of EMDR therapy—such as stabilization, resourcing, desensitization, reprocessing, and forming beliefs about the self and future—into a cohesive program. This paper aims to introduce the PIPA Program and discuss the outcomes of this program in reducing distress and facilitating trauma reprocessing with a group of more than 220 traumatized individuals in Ethiopia.

While EMDR therapy is typically administered in individual settings by trained mental health practitioners, a growing body of literature has shown its efficacy in group settings (3, 4) while also addressing the safety concerns associated with potential risk of simultaneous abreactions within a large group. In response to the aftermath of Hurricane Pauline on the western coast of Mexico in 1999, Jarero et al. (5) developed and implemented the EMDR-Integrative Group Protocol (EMDR-IGTP) (6) to both adults and children affected by the disaster. This protocol has since been implemented globally, in both its original and adapted forms, for diverse populations of children and adults (7–9). The development of the Integrative Group Trauma Protocol (IGTP) by Jarero, Artigas, and Hartung (3) in 2006 demonstrated the feasibility of reprocessing critical incidents within group settings. Other EMDR therapy group interventions have emerged, such as the Group Traumatic Episode Protocol (G-TEP; 4) and the Flash Technique for groups (10, 11) among others. These innovations have facilitated the ongoing expansion and development of group-oriented therapeutic approaches.

As a response to the 2019 Global Initiative for Stress and Trauma Treatment (GIST-T) (https://gist-t.org/projects/#past), which aimed to create scalable programs capable of reaching a broader population through the utilization of group protocols, Carvalho and Guz developed the PIPA Program which is suitable for implementation by both EMDR and non-EMDR trained therapists in group settings. Unlike other stand-alone EMDR therapy group protocols that focus on a single aspect of treatment, the PIPA Program integrates the various standalone exercises into an intervention program. Thus, the main goal of the PIPA Program’s development was to organize low-intensity group exercises into one cohesive structure. A secondary goal was to create a program that could not only be implemented in crisis interventions and disaster situations but could also be used in ongoing emotional growth group settings, such as divorce recovery, self-esteem, grief and loss, etc. A significant advantage of the PIPA Program is its adaptability for use with large groups, a feature not always feasible with some group interventions that require a high facilitator-to-participant ratio.

The PIPA Program combines three group stand-alone exercises: 1) “Pillars of Life,” an exercise originally conceived by Raimundo (12) and modified for EMDR processing (7) 2) the Flash Technique (FT), created by Manfield et al. (13) and later adapted for group settings by Sik-Lam Wong (10); and 3) the “Quadrants” exercise, Carvalho’s (2022) simplification of the EMDR Integrative Group Treatment Protocol for Adults (14). These exercises form the core of the PIPA program.

In 2021, the PIPA Program underwent initial pilot testing in the United States in English with a diverse international cohort of 12 participants, represented by six language groups: English, Portuguese, Spanish, German, Cantonese, and Mandarin. This pilot aimed at refining the program based on the outcomes observed. A subsequent pilot in Brazil with a larger group of EMDR therapists yielded positive participant feedback.

## 2 Methods

### 2.1 Procedure

NGOs and corporate companies brought together individuals for the PIPA Program gatherings, which were conducted over two to three days. On the first day, the PIPA leader trained local psychologists and health professionals in stabilization tools for participant support. These psychologists acted as part of the support team helpers for the PIPA Program. The PIPA Programs were delivered in either two or three days. Day-1 focused on psychoeducation, including topics such as trauma’s impact and its consequences, stabilization exercises such as abdominal breathing and progressive muscle relaxation, and fostering a therapeutic group rapport. The Pillars of Life exercise was introduced on Day-1 in the two-day format and on Day-2 in the three-day format. The Group Flash Technique and the Quadrants exercise were both administered on the final day. During the group exercises, personal experiences were generally not shared with the exception of the positive resources elicited from the Pillars of Life. Participants completed the PCL-5 and ACE questionnaires on the first day (pre-test) and last day (post-test). The SUDS were recorded during the Group Flash Technique and after each drawing in the Quadrants exercise.

The PIPA Program was administered in English, as all education in Ethiopia is conducted in this language. Although most Ethiopians understand English well, their ability to speak or write it fluently can vary. To address any language difficulties, the facilitator, a native Amharic speaker, was available to clarify questions in Amharic. Additionally, a translator for Tigrinya was present during group sessions as needed.

Trust and rapport were important considerations because of tribal and language differences. Notably, the PIPA leader belonged to an opposing faction in the war, adding to the initial distrust. One adaptation to the Pillars of Life exercise was to have group participants share their associated positive experiences. After participants identified three or four positive memories they paired up and shared their drawings. This lasted approximately 30 minutes. After this, the group was divided into two, where each group observed and described the drawings of the other group. The purpose of this sharing and presenting drawings and the stories behind them was to foster a sense of rapport, engagement, and group cohesion.

### 2.2 PIPA program group exercises

In the comprehensive framework of the PIPA Program, each protocol serves a unique and crucial function. The Pillars of Life (7) functions as a resource installation, encouraging participants to recall and focus on the positive and beautiful aspects of their lives. The Flash Technique (13) plays a pivotal role in safely desensitizing traumatic and stressful memories. Its unique approach allows group members to address both recent and past traumas without having to endure their associated emotional distress. The Quadrants exercise is designed for the direct reprocessing of traumatic memories or experiences, and to look to the future. Finally, a concluding round using the Flash Technique (FT) is designed to help further alleviate any remaining distress, ensuring that participants can leave the meeting with a sense of closure and reduced emotional burden. The following sections describe each protocol in more detail.

#### 2.2.1 The Pillars of Life 

The Pillars of Life technique, conceived by Raimundo (1982 - or ref 15) and later adapted by Carvalho for EMDR therapy, is designed to bolster resilience (7) and well-being. This method focuses on identifying and reinforcing positive life experiences, facilitating personal growth and resilience (11). In this exercise, participants are provided with a timeline worksheet, marked with “birth” and “today” to represent their life’s journey. They are invited to recall and symbolically depict positive events or influential persons from their past on this timeline, correlating with the age at which these memories occurred. These positive recollections, encompassing supportive relationships, achievements, and memorable experiences, are then explored to elicit associated positive beliefs, emotions, and physical sensations. Participants were encouraged to articulate “words of wisdom”, or advice derived from these experiences, and to further consolidate and reinforce the memory with bilateral movements like self-hugs or leg tapping.

#### 2.2.2 The Flash Technique

The Flash Technique (FT) originally developed by Manfield et al. (13) represents a significant advancement in EMDR therapy, particularly in its Preparation and Stabilization Phase. Initially designed for individual clients, its primary objective is to mitigate the emotional intensity of distressing memories, thereby maintaining the client within their window of tolerance for the subsequent phases of EMDR reprocessing. Over time, FT has evolved into a standalone desensitization and reprocessing technique, demonstrating remarkable efficacy not only in individual settings but also in group contexts.

Wong (10) and Manfield et al. (11) have been pivotal in adapting and validating the use of FT in diverse group settings. Wong’s adaptation was successfully applied in a men’s shelter with substance abusers as early as December 2017, demonstrating large trauma symptom reduction with 8 sessions in a small group of 5 individuals. Manfield extended the application of FT to healthcare groups of up to 40 individuals, showing substantial reduction in subjective level of disturbance in a single session.

FT groups also developed independently outside the US. Yaşar et al. (16, 17) demonstrated in two studies that a single session of FT in a group format could result in substantial reduction in trauma symptoms. Yasar’s 2021 study also demonstrated continued improvement 30 days after the FT group.

Furthermore, a randomized control trial by Yaşar et al. (18) compared FT with a World Health Organization stress management module (Improving Mental Health Training for Primary Care Residents; mhGAP) in a group of people traumatized by traffic accidents and showed that FT was a superior trauma intervention.

A distinctive feature of the Flash Technique is its ability to reduce distress without requiring the individual to vividly recall or emotionally engage with the traumatic memory (19). This safety aspect is particularly beneficial in group settings, where resources, time, and facilitator attention may be limited. In practice, the technique involves the identification of a Positive Engaging Focus (PEF)—a distraction that is enjoyable and easy to concentrate on, such as a pleasant memory, a hobby, or a neutral activity like mindful breathing. Participants have relayed that their PEF involved activities such as singing, counting numbers, creating art, thinking of one’s pets, past trips, sunsets or watching an engaging video. Simultaneously, the participant briefly acknowledges the distressing memory and then mentally sets it aside, often visualizing placing it in a ‘healing box’ out of sight. The participants are then instructed to focus *exclusively* on the PEF and not connect with the disturbing memory.

With the project in Ethiopia, participants were offered specific suggestions as a positive engaging focus, such as eating their favorite food, watching a beautiful sunset, wearing their favorite piece of clothing, remembering a trip or a visit that had been a good experience, as an additional layer of safety, to help them avoid connecting with previous painful memories. If individuals did not like the suggested PEF’s (Positive Engagement Focus) they were free to create one for themselves. Once the PEF had been established, the therapist prompted the individual to quickly blink their eyes three times when they were given a cue word such as “flash” or “blink” while doing a form of slow tactile bilateral stimulation (self-hugs or tapping on their legs) all the time thinking of the positive focus. After a few sets of blinks, the therapist asked the person to check in delicately) and “have a peek from far away at the memory you put in the ‘healing box’”) with the disturbing memory to see if there were any changes. The therapist repeated the rounds of blinking and checked in with the memory a total of five times, subsequently asking if anything different was noticed about the memory, and to rate the present level of disturbance of the memory (SUDS) as they “peeked from afar”. Usually, the person noticed a reduction in the level of disturbance without consciously trying to process the memory and without any awareness of what happened during the process.

#### 2.2.3 The Quadrants

The Integrated Group Treatment Protocol (IGTP), the precursor to the Quadrants exercise in the PIPA Program, has been effective in the processing of trauma-related emotional distress among large groups (3, 14). The Quadrants exercise, simplified and adapted for the PIPA Program, included assigning a title to each quadrant, and fewer initial steps. The Quadrants Exercise involves participants folding a page into four sections. In the first quadrant, they depict the distressing event, assign a title, and rate their Subjective Units of Distress (SUDS). Tactile bilateral stimulation, such as self-hugs or tapping, accompanies this and each subsequent quadrant. This cycle of drawing, titling, SUDS measurement, and bilateral movements is replicated in each quadrant. Upon completion, participants envision their future selves on the reverse of the sheet and note their final SUDS score. The exercise concludes with a brief body scan to identify sensations, followed by short bilateral movements. Participants are then given the opportunity to ask questions, finalizing the exercise.

### 2.3 Instruments

The Posttraumatic Stress Disorder Checklist for *DSM-5* (PCL-5; 20) and the Adverse Childhood Experiences Scale (ACE; 21) were administered at the beginning of the PIPA Program. The PCL-5 was re-administered after participants completed the PIPA program. The PCL-5 is a 20-item self-report measure that assesses DSM-5 symptoms of PTSD. Individuals were asked to rate items on this scale from 0 (not at all) to 4 (very true). Participants were instructed to complete the Post-Treatment PCL-5 with the memories they were thinking about when they filled out the Pre-Treatment PCL-5. An ACE score represents a cumulative count of various forms of abuse, neglect, and other indicators of a challenging childhood. Individuals are asked to check up to 10 items that occurred before they were 18 years old. The items are not rated for frequency or severity.

Subjective Units of Disturbance Scale (SUDS) assesses the subjective intensity of disturbance or distress currently experienced by an individual on a scale ranging from 0 (nothing, no disturbance) to 10 (extremely high). SUDS were collected for pre- and post-intervention of the first and second round of the Flash Technique and for the Quadrants.

### 2.4 Participants

A PIPA-trained EMDR therapist administered the PIPA Program to more than 220 individuals (average age between 30-39 years old), most of whom had been living in a war for two years (2020-2022). They reported traumatic experiences which included extreme food scarcity, lack of medical care, disrupted communication services, physical injuries, loss of family and friends, witnessing or undergoing severe traumatic events, displacement, and knowledge of acquaintances who had been raped or injured. Some had directly participated in the conflict.

All participants were college graduates, with 86% of them living in Tigray and 14% living in other areas of the country. Participants were predominantly male (95%) and had resumed their pre-war employment. A PIPA-trained EMDR therapist administered the protocols to groups that ranged from 35 to 50 participants. The primary focus of these groups was on treatment, and the secondary focus was on research, therefore there was no exclusionary screening.

## 3 Results

About 90% of the participants completed the initial PCL-5, ACE, and SUDS scales. A range of 188 to 198 participants completed various components of the PIPA program and were included for analysis. Two women received extra individualized stabilization from a PIPA support person and returned to the group exercises. Although the Pillars of Life exercise did not involve the measurement of SUDS, a noticeable improvement in the participants’ mood following the exercise was observed by the leader. Group members came to the event withdrawn and disconnected. As this exercise progressed, they began to exhibit more positive behaviors, such as smiling and engaging in communication. The overall outcomes of the various segments within the PIPA Program, along with the pre-and post-treatment scores on the PTSD Checklist (PCL-5), are summarized below on **Table 1**.

**Table 1:** Pre- and post-treatment distribution of frequencies of PCL-5 scores.

PCL-5 Score	Pre-Treatment *N*	Post-Treatment *N*
0 to 20	17	107
21 to 32	44	45
33 and above	127	36

Note a PCL-5 score of 33 would indicate likely PTSD diagnosis.

For the PCL-5 surveys, with an N = 188, the mean pre-treatment score was 38.58 (SD = 14.91, 95% C.I. = [3.645, 40.71]). The post-treatment score was 20.59 (SD = 13.99, 95% C.I. = 18.59, 22.59). The Cohen’s d was 1.244 showing a large effect size. The p-value (2 tails) was <0.00001 showing statistical significance. The number of participants likely to meet the criteria for a PTSD diagnosis (cut off score of 33) decreased from 127 (67.55%) to 36 (19.15%) in the pre- and post-treatment evaluations.

The ACE scale was completed by 198 individuals who reported a mean of 2.11 (SD = 2.079, 95% C. I. = [1.821, 2.401]) potentially traumatic childhood events. As a comparison, more than half of Felitti et al.’s (21) 17,000 respondents reported at least one ACE score, and one-fourth reported more than two categories of adverse childhood exposures. In addition, in a small sample of six individuals in a men’s shelter for substance abusers, the mean ACE score was 6.2 (SD = 2.68) (10). We are not aware of any published ACE data of a comparable population for Ethiopia for comparison.

For the first Flash Technique segment, with N = 197, the mean pre-treatment SUDS score was 8.523 (SD = 2.327, 95% C.I. = [8.198, 8.848]). The mean post-treatment SUDS score was 1.264 (SD = 2.249, 95% C.I. = [0.95, 1.578]). The Cohen’s d was 3.877 which shows a large effect size. The 2-tailed p value was <0.00001 which shows a statistically significant difference between pre- and post-treatment scores.

Similarly, for the Quadrants segment, with an N = 193, the mean pre-treatment SUD score was 8.42 (SD = 2.132, 95% C.I. = [8.119, 8.721]). The mean post-treatment SUDS score was 1.611 (SD = 2.216, 95% CI = [1.298, 1.924]). Cohen’s d was 3.131 which shows a large effect size. The 2-tailed p value was <0.00001 which shows a statistically significant difference between pre- and post-treatment scores.

**Figure 1:**
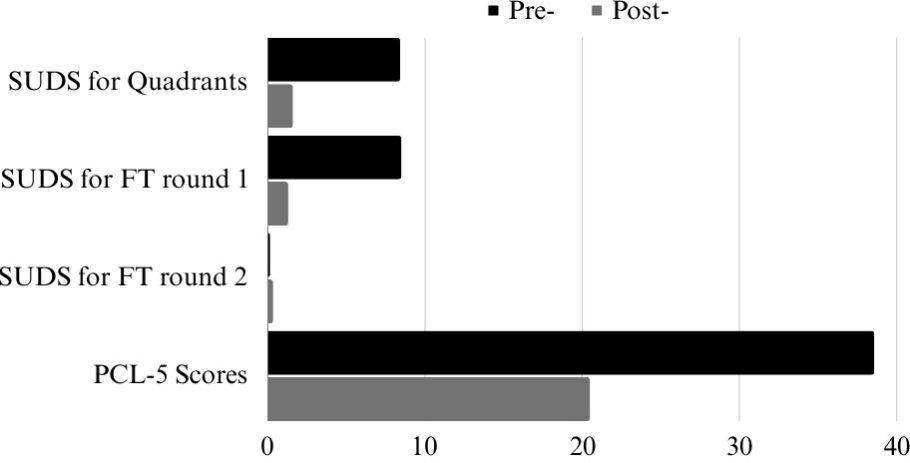
Mean pre- and post-treatment for Quadrands, Round 1 & 2 of Flash Technique (FT), and PCL-5 Scores of Ethiopian sample.

For the second Flash Technique segment, with an N = 188, the mean pre-treatment SUD score was 0.154 (SD = 0.985, 95% C.I. = [0.013, 0.295]). The mean post-treatment SUD score was 0.346 (SD = 1.289, 95% C.I. = [0.162, 0.346]). Cohen’s d was 0.167 showing a small effect size. The 2-tailed p value was < 0.029 which shows a statistically significant difference between pre- and post-treatment scores. See **Table 2** and **Figure 1**.

**Table 2:** Mean pre- and post-treatment PCL-5 and SUDS for 1^st^ and 2^nd^ FT, and Quadrants of Ethiopian sample.

	Pre-Treatment	Post-Treatment	p-value (2 tails)	Cohen’s d
FT first segment N=197	8.523 (SD=2.327, 95% CI= 8.198, 8.848)	1.264 (SD=2.249, 95% CI= 0.950, 1.578)	<0.00001	3.877
Quadrants N=193	8.42 (SD=2.132, 95%CI= 8.119, 8.721)	1.611 (SD=2.216, 95% CI= 1.298, 1.924)	<0.00001	3.131
FT second segment N=188	0.154 (SD=0.985, 95% CI=0.013, 0.295)	0.346 (SD)=1.289 95% CI=0.162, 0.346)	<0.029	0.167
PCL-5 N=188	38.58 (SD=14.91, 95% CI= 36.45, 40.71)	20.59 (SD=13.66, 95% CI= 18.59, 22.59)	<0.00001	1.244

**Link to data:** Data from Ethiopia project 2023.

## 4 Discussion

The results of this study suggest that the PIPA program is safe and effective and can be used with severely traumatized populations with significant positive results. The program had a high completion rate; no attendees experienced severe abreactions, and only two participants, out of more than 220 participants, needed brief individual interventions before returning to the program.

Our data shows that attendees processed their disturbing memories with the Flash Technique and Quadrants resulting in a statistically significant improvement of PTSD symptoms and on levels of distress before and after they completed the PIPA program.

The PIPA Program was designed to loosely follow the standard EMDR eight phases. In the PIPA Program, each participant was taught self-regulation exercises and completed the Pillars of Life exercise which corresponded with the preparation and stabilization phase. The Flash Technique was used to reduce emotional disturbances, facilitate desensitization, and set the stage for further processing in the Quadrants exercise. Once the memory was considerably desensitized with the Flash Technique, then participants moved toward fully reprocessing that disturbing memory directly with the Quadrants exercise. The Quadrants exercise was structured to address past traumas and lead participants in thinking about the future. The data, however, suggested that participants processed two different disturbing memories, one with the Flash Technique and the other with the Quadrants. The Flash Technique was applied again so that participants could effectively manage and compartmentalize any residual emotional disturbances and leave the session in a psychologically regulated and stable state. This exercise limited the emergence of new traumatic memories during the concluding stages.

The PCL-5 (PTSD Checklist for DSM-5) is a standardized self-report rating scale for PTSD symptoms, and in this context, it was used to measure the effectiveness of the PIPA Program’s intervention on the participants’ PTSD symptoms related to specific traumatic memories. The ACE scores in this sample were surprisingly low (ACE M = 2.11). Yet, participants in our sample reported a high level of PTSD symptoms. This suggests that PTSD symptoms are likely to stem from experiences of war and not necessarily from adverse childhood events. Participants were instructed to complete the Post-Treatment PCL-5, focusing on the same memories they had in mind during the Pre-Treatment PCL-5. This was done to assess the impact of the PIPA Program on specific traumatic memories. The pre- and post-treatment PCL-5 data indicated a significant shift in the likelihood of a PTSD diagnosis among participants. The results suggested improvements in symptoms such as hyperarousal, intrusion, avoidance, and affect.

The data showed that the FT is effective in desensitizing traumatic memories. This is shown by significant drops in SUDS scores post-Flash Technique and post-Quadrants, indicating that these exercises were effective forms of desensitization and reprocessing of traumatic memories. The SUDS for the first round of FT showed an average reduction of about seven points which was statistically significant and clinically meaningful. The average pre- and post FT SUDS of the second round fell well below one, suggesting that the reduction of distress levels attained during the previous FT were maintained.

The PIPA leader slowly built an empathic alliance with the participants to create trust. Substantial changes were observed in the participants’ demeanor after the Pillars of Life segment by the therapist. Instead of being withdrawn as they had been at the start of the meetings, attendees were smiling and communicative after this part of the program and developed a more positive attitude. No measures were taken for the Pillars of Life exercise since it was a resource installation, aimed at helping participants self-regulate within or outside of the group context. Many indicated that they were planning to do the Flash Technique on their own for self-care post-PIPA, and some had already begun to do so.

The program’s ability to facilitate processing of multiple traumatic memories in a safe and effective manner is significant, especially given the high PTSD likelihood among participants. This method’s success in a non-clinical, real-world setting with severe trauma suggests its potential applicability in various traumatic contexts. The positive post-treatment shifts in PCL-5 scores imply the program’s potential in reducing PTSD symptoms.

### 4.1 Limitations and recommendations

Despite a high completion rate, several factors influenced the program’s implementation and data collection, shedding light on the complexities of administering trauma-focused interventions in diverse and distressed populations. A small number of participants did not complete all the scales. A variety of factors including fatigue, lack of trust, and unidentified or unforeseen issues may be responsible for these incomplete results. It is also possible that the severely traumatic nature of the participant’s experiences resulted in some participants’ hesitation to fill out some of the worksheets.

The program was implemented as a one-time intervention, not an ongoing recovery process, limiting insights into its long-term efficacy. This was the first time that the PIPA Program was used with a severely traumatized population. Although this is an important component for intervention-focused research, it was not possible to complete the follow-up given the restrictions of leaders and the significant geographical distribution of the population who participated in the PIPA Program. We did not quantify the effect of the stabilization and Pillars of Life segments. It was not anticipated the participants’ moods would be as strongly impacted by these exercises as they were, since it was originally included as a resource installation. The PCL-5 data may not indicate a global reduction in PTSD symptoms. It is noted that the participants might have more disturbing memories that need processing in the future, which aren’t captured in the current PCL-5 assessment.

Future research should address the identified limitations and explore long-term effectiveness and broader applicability. Future iterations of the PIPA Program should consider incorporating quantitative measures for all segments, including the Pillars of Life exercise. Long-term follow-up studies are crucial to understand the lasting impact of the program and to validate its effectiveness over time. Exploring the PIPA Program as an ongoing recovery tool and its adaptability across different therapeutic contexts would be beneficial for broader application.

## 5 Conclusions

Our data shows that the PIPA Program was safe and effective in processing traumatic memories among a severely traumatized population in Ethiopia. The PIPA Program was successfully implemented in a non-clinical setting and shows potential applicability in various contexts, especially where individualized PTSD treatments may not be feasible. It also shows promise in being a culturally sensitive and adaptable program in working with diverse populations who present with traumatic symptoms. -

## Data availability statement

The original contributions presented in the study are included in the article/supplementary material. Further inquiries can be directed to the corresponding author.

## Ethics statement

Ethical approval was not required for the study involving humans in accordance with the local legislation and institutional requirements. Written informed consent to participate in this study was not required from the participants or the participants’ legal guardians/next of kin in accordance with the national legislation and the institutional requirements.

## Author contributions

SW: Investigation, Project administration, Resources, Writing – review & editing. DA: Funding acquisition, Project administration, Resources, Supervision, Validation, Writing – review & editing. EC: Conceptualization, Methodology, Software, Writing – original draft, Writing – review & editing, Resources. SW: Formal analysis, Writing – original draft, Writing – review & editing, Validation. RH: Conceptualization, Writing – review & editing.
